# Exploring the Interfacial Phase and π–π Stacking in Aligned Carbon Nanotube/Polyimide Nanocomposites

**DOI:** 10.3390/nano10061158

**Published:** 2020-06-12

**Authors:** Qian Jiang, Qian Zhang, Xianyan Wu, Liwei Wu, Jia-Horng Lin

**Affiliations:** 1 Tianjin and Ministry of Education Key Laboratory for Advanced Textile Composite Materials, Tiangong University, Tianjin 300387, China; jiangqian@tiangong.edu.cn (Q.J.); zhangqian_19959@163.com (Q.Z.); 2 Innovation Platform of Intelligent and Energy-Saving Textiles, School of Textile Science and Engineering, Tiangong University, Tianjin 300387, China; 3 Key Laboratory of Yarn Materials Forming and Composite Processing Technology of Zhejiang Province, College of Material and Textile Engineering, Jiaxing University, Jiaxing 314001, China; 4 Laboratory of Fiber Application and Manufacturing, Department of Fiber and Composite Materials, Feng Chia University, Taichung 40724, Taiwan

**Keywords:** polyimide (PI), multi-walled carbon nanotubes (MWNT), nanoscale, interface, π–π stacking

## Abstract

To characterize the interfacial microstructure and interaction at a nanoscale has a significant meaning for the interface improvement of the nanocomposites. In this study, the interfacial microstructure and features of aligned multiwalled carbon nanotube (MWNT) and conjugated polymer polyimide (PI) with three molecular structures were investigated using small-angle X-ray scattering (SAXS), wide-angle X-ray diffraction (WAXD), and fluorescence emission spectroscopy. It was found that aligned MWNT/PI nanocomposites had a nonideal two-phase system with the interfaces belonging to long period stacking ordered structure. Attributed to the π–π stacking effect, MWNT/BTDA-MPD presented the most regular arrangement verified by fractal dimension. By adopting a one-dimension correlation function, each phase dimension in aligned MWNT/PI nanocomposites was calculated and verified by high resolution transmission electron microscopy (HRTEM) and X-ray diffraction (XRD). The π–π stacking was demonstrated to be an important interaction between MWNT and PI via WAXD and fluorescence emission spectroscopy, and it was influenced by the linkage bond between benzene rings in PIs. This work is of significance to reveal the interfacial features between conjugated polymer and carbon nanotubes (CNTs), which is favorable for the interface design of CNT-based high performance nanocomposites.

## 1. Introduction

Carbon-nanotube (CNT)-based nanocomposites have engaged much attention in recent years due to their extraordinary performance [1,2,3]. Carbon nanotubes are primarily composed of C–C bonds that are attributed to the sp2 hybridization, and thus have a one-dimensional special structure, presenting unique properties, such as favorable mechanical properties, good flexibility, and better electrical conductivity. Hence, carbon nanotubes are an ideal reinforcement for nanocomposites, and commonly used in a variety of applications, such as in sensors [4], actuators [5], capacitors [6], and hydrogen storage materials [7]. The application of nanocomposites strongly depends on the challenge of how to deal with the interface characteristics between CNTs and the matrix [8,9,10].

Featuring excellent thermal stability, irradiation resistance and dielectric properties, polyimide (PI) has been pervasively used in the aerospace, aviation, and electronic fields [11,12]. PI/CNT nanocomposites, rather than pure PI matrix, show greater mechanical strength, Young’s modulus, electrical conductivity, and thermal stability [13,14]. However, the mechanical properties of the nanocomposites are still far behind the values predicted by the rule of mixtures. The superiority of the mechanical properties of nanotubes alone does not ensure mechanically superior nanocomposites, because the interfacial properties between carbon nanotube and polyimide are one major influential factor to the nanocomposite properties [10]. In our previous experimental and simulation works, it has been found that, by using the aligned CNT, the strength of the carbon nanotube can be utilized to a large extent in CNT/polyimide nanocomposite [15,16]. What’s more, according to the molecular dynamics simulation, the π–π stacking effect dominates in the interfacial region of CNT/polyimide nanocomposite [17,18].

CNT and conjugated polymers containing the structure of aromatic ring show remarkably different interface characteristics due to the noncovalent interactions of the interface, such as electrostatic interaction, van der Waals force, and π–π stacking effect [19,20,21,22]. By means of π–π stacking, the surface modification of carbon nanotubes can be conducted [23]. The micro-sized polystyrene/multiwalled carbon nanotube (PS/MWNT) particles can be coated by boron nitride (h-BN) completely via the π–π stacking, which in turn generated good conductivity [24]. Serving as a driving force, π–π stacking modified carbon nanotubes, which provided novel nanotube materials with broad prospects as in new dimension, shape, and potential property [25]. Besides, π–π stacking can control multi-walled carbon nanotubes (MWNTs) in the interface of polystyrene/poly(methyl methacrylate) (PS/PMMA) composites [26]. 

Although previous studies indicated that π–π stacking influenced the interaction between the conjugated polymer and carbon nanotubes, there are few studies on that explain how it influences or that give detailed interfacial characterization. In order to explore the interfacial interaction between CNT and conjugated polymers, three polyimides with different structure were selected as representatives in this work. The reason for choosing these three polyimides is the typical functional groups in diamine and dianhydride play an important role in deciding the benzene ring alignment, thus influencing the π–π stacking and interfacial interaction. The interface features of aligned MWNT/PI nanocomposites, especially π–π stacking of the interface were characterized using wide-angle x-ray diffraction (WAXD), small-angle X-ray scattering (SAXS) and fluorescence emission spectroscopy, thermal field emission scanning electron microscope (FESEM), and high-resolution transmission electron microscopy (HRTEM). This work is of significance to reveal the interfacial features between conjugated polymer and CNTs, which is favorable for the interface design of CNT-based high performance nanocomposites.

## 2. Materials and Experiments

### 2.1. Fabrication of the Aligned Carbon Nanotube/Polymide Composites

**Figure 1:**
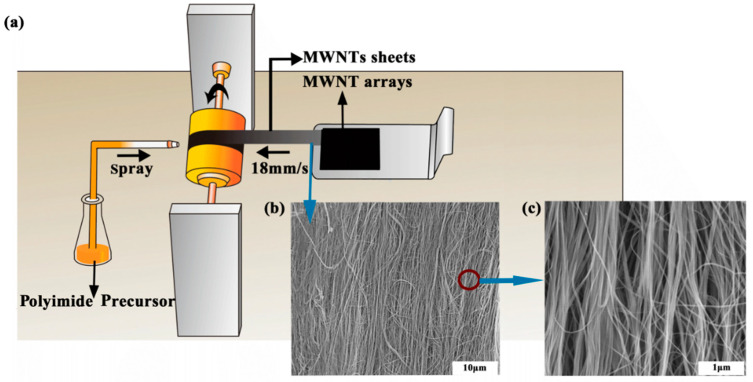
(**a**) Fabrication of the aligned multiwalled nanotube/polyimide (MWNT/PI) nanocomposites using the spray winding method. (**b**,**c**) Scanning electron microscope (SEM) images of aligned carbon nanotubes (CNTs) in nanocomposite.

**Figure 2:**
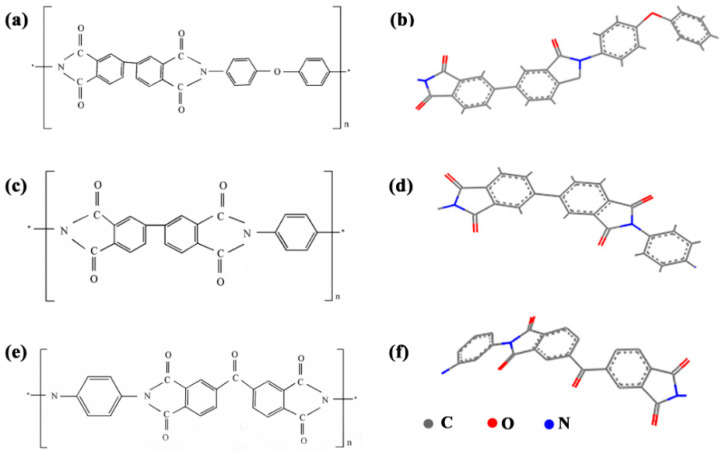
Three types of polyimides with different molecular structures. (**a**,**b**) BPDA-ODA, (**c**,**d**) BPDA-PDA, (**e**,**f**) BTDA-MPD.

The MWNT array used to produce aligned MWNT/PI composite was grown using chemical vapor deposition (CVD) [15,16] with a height of 700 μm, a wall thickness of 5–6 nm and a diameter of 10 nm. The MWNT ribbons were pulled out from MWNT array at a speed of 18 mm/s and wound onto a rotating cylindrical polytetrafluoroethylene (PTFE) spool. During the winding process, the polyimide precursor in the form of polyamic acid diluted to 0.1 wt% by *N*-methyl-2-pyrrolidone (NMP) was sprayed onto the MWNT ribbons layer by layer (Figure 1). The NMP was used as the original solvent and it was proved to have good compatibility with 3,3′,4,4′-biphenyltetracarboxylic dianhydride-4,4′-diaminodiphenyl ether (BPDA-ODA), Poly(biphenyl dianhydride-p-phenylenediamine) (BPDA-PDA) and benzophe-nonedianhydride-m-phenylenediamine (BTDA-MPD) polyamic acids [15,16]. The chemical structures of three polyimides were presented in Figure 2, and Figure 2b,d,f was sketched in Materials Studio 7.0 and optimized to chemically reasonable using ‘Clean’ function. The BPDA-ODA polyimide precursor (Ube Industries, Ltd., Tokyo, Japan) has the Mw and Mn of 31,000 and 16,000. The BPDA-PDA polyimide precursor (Ube Industries, Ltd., Japan) has the Mw and Mn of 26,000 and 14,000. The BTDA-MPD polyimide precursor (Sigma-Aldrich, Co. LLC., Shanghai, China) has the Mw and Mn of 28,000 and 16,000. The nanocomposite fabricated by the spray winding method was 30 μm thickness after 2 h of winding, and was then hot-pressed in a vacuum oven at 120 °C for 2 h to evaporate the solvent and make multiwalled nanotube/polyimide (MWNT/PI) composites more compact. The curing process followed a stepwise heating program from 120 °C to 450 °C.

### 2.2. Characterizations

The synchrotron small-angle X-ray scattering (SAXS) was conducted in Shanghai Synchrotron Radiation Facility (BL16B1, France) with an energy of 10 KeV. The wavelength of the X-ray was 0.124 nm. This distance between the sample and the detector (PiLatus 2 M) was 1921 mm. Sample dimensions were 253.7 mm × 288.8 mm, and the pixel size was 172 μm × 172 μm. The thickness of composites was 30 μm and the incident X-ray was projected along the thickness direction. Fit2d software (European Synchrotron Radiation Facility, France) was used to digitize the SAXS image and analyze the data. 

The wide-angle X-ray diffraction (WAXD) (Pilatus 3R 200 K, Xenocs, Francde, France) was used for the interfacial emulation. The wavelength of the X-ray was 0.124 nm. The pixel size was 172 μm × 172 μm. The light source output power was 50 kV and 0.6 mA. 

The fluorescence emission spectra were recorded on an F-74600 fluorescence emission spectrometer (F-4600, Hitachi, Japan) at the fluorescence emission wavelength of 250 nm and the pixel size was 1 cm × 1 cm. Samples were placed in a solid sample holder that was then placed in a fluorometer with excitation wavelengths 250 nm. The sample was then screened at a rate of 1200 nm/min with a PMT voltage of 500 V.

The thermal field emission scanning electron microscope (FESEM, Gemini SEM500, Carl Zeiss AG, Oberkochen, Germany) was applied to observe the morphology of MWNT/PI nanocomposites. Samples were processed with an ultrathin section method. The interface of the nanocomposite was observed via high-resolution transmission electron microscopy (HRTEM, JEOL JEM-2100F, Carl Zeiss AG, Germany).

The X-ray diffraction (XRD) was tested on an X-ray diffractometer (D8 Discover, Bruker, Karlsruhe, Germany). The X-ray source was CuKα (λ = 0.15418 nm), and the test temperature was room temperature. The tube voltage was 40 kV, the tube current was 40 mA, and the scanning range was 5°–50°.

## 3. Results and Discussions

### 3.1. Interface Characteristics of MWNT/PI Nanocomposite

**Figure 3:**
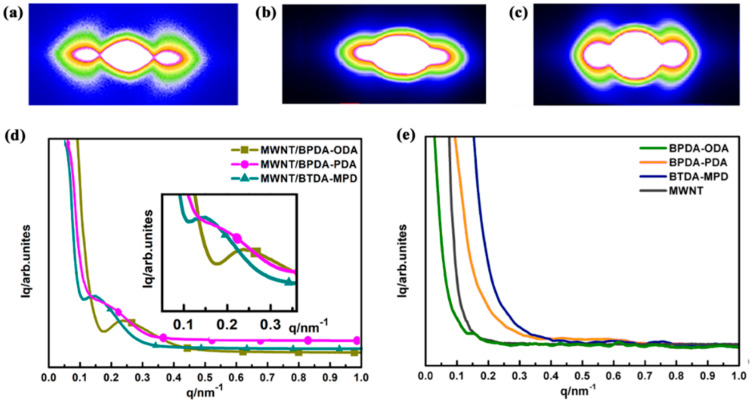
The small-angle X-ray scattering (SAXS) curves of (**a**) BPDA-ODA, (**b**) BPDA-PDA, and (**c**) BTDA-MPD. SAXS intensity as a function of scattering vector for (**d**) aligned carbon nanotube/polyimide composites and (**e**) PIs and MWNTs.

SAXS demonstrates the coherent scatter within a small angle range of 2°–5° that is close to an elementary beam when the X-ray penetrates a sample. SAXS originates from spatial fluctuations of the electronic density within a material. It is ideally suitable for investigating the geometric structure of inhomogeneous material [27,28]. By computing and analyzing the scattering curves, the shape, size, distribution, and content of microstructure can be obtained, and then the interfacial features and the fractal characteristics are deduced [29,30]. After using Fit2d software to reduce the subtracting air scatter, the background intensity was rectified to make up for the visual clarity, thereby obtaining the two-dimensional SAXS scattering diagram. Figure 3a–c shows the dumbbell type in the scattering diagram, indicating that the three MWNT/PI nanocomposites have anisotropic long-period structures. 

By using the integral along the equatorial directions, the relationship between the scattering intensity (Iq) and scattering vector (q) is presented in Figure 3d,e. In Figure 3d, with an increase in the scattering vector, the scattering intensity decreases sharply while single shoulder peaks are presented between 0.1–0.4 for three MWNT/PI nanocomposites, indicating there is a long period stacking ordered structure. In order to examine the specific scatterer causing the long-period signals, phonon dispersion curves of pure PIs and CNT were plotted in Figure 3e. It is exhibited that the shoulder peaks are absent in curves of both PIs and CNTs, indicating that the scatterer that causes the long-period signals is attributed to the interface between CNT and PI.

**Figure 4:**
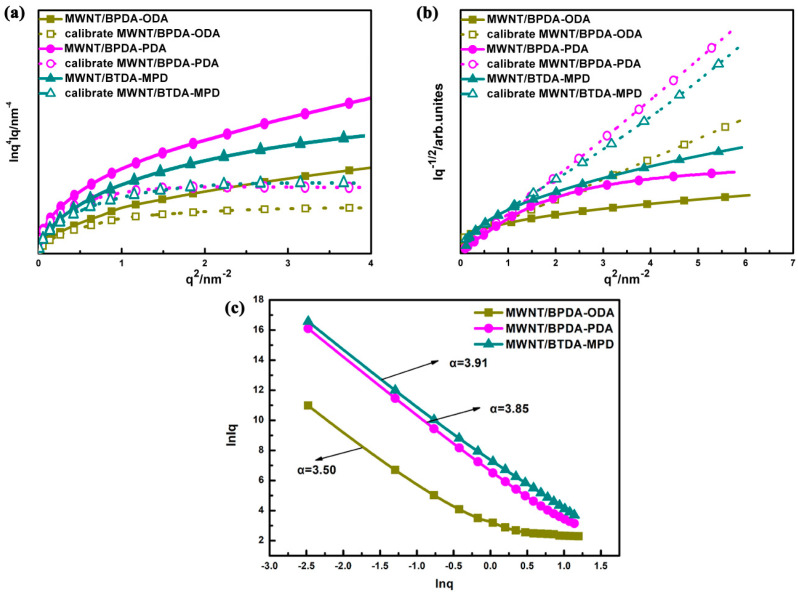
Porod-law analysis (**a**) and Deybe-law analysis (**b**) of scattering curves of the aligned MWNT/PI nanocomposites with actual plots as solid line and calibrated plots as dashed line. (**c**) Double logarithmic curve of the aligned MWNT/PI nanocomposites.

The SAXS scattering curves of three MWNT/PI nanocomposites reveal that there are nanoscale scatters with uneven electron density. Therefore, the Porod law was used to determine whether the interface between two phases is sharp. According to the Porod law [31], within the range of higher angle region, if the interface is sharp, the product of *I*(*q*) and *q*^4^ is close to the *K* constant based on Equation (1). *K* is named as the Porod constant, which corresponds to the sharp interface where uniform electron density is presented. In Figure 4a, the curve has a positive slope (i.e., positive deviation) in a high-angle area, indicating the presence of a nonideal two-phase system. The individual electron density (i.e., ρ1 and ρ2) of two phases displays fluctuation, instead of mutation, which suggests that a diffuse interface exists between the two phases. The dashed curves in Figure 4a are the Porod curves (Equation (2)) calibrated to eliminate positive deviation. In this way, it can be also proved that all of the three nanocomposites have a nonideal two-phase system.

(1) $$\underset{q\to \infty }{\lim}\ln[q^{4}I(q)]=\ln K$$

(2) $$I{\left(q\right)}^{\prime }=\exp(-bq^{2})*I(q)$$

where $I{\left(q\right)}^{\prime }$ is the calibrated scattering intensity and b is a relative parameter to the fluctuation of additional electron density.

Based on the Debye law [32], the correlation of electron density fluctuation between two arbitrary scatters can be obtained, which can be used as a reference for whether the scattering system is ideal as well as to compute the correlation distance (*Ac*). Based on the scattering curves of Figure 4b, the curves are not linearly plotted based on Debye law. Instead, a significant curve behavior is observed in the measured range, which, once again, proves that all of the three nanocomposites have a nonideal two-phase system. By fitting the Debye plot with Equation (3), which stands for the ideal two-phase system, the correlation distance (*Ac*) can be calculated. The three nanocomposites have a correlation distance of 0.43 nm, 0.31 nm, and 0.16 nm, demonstrating that within the specific range, nanoscale scatters are strongly correlated.

(3) $$I{\left(q\right)}^{-\frac{1}{2}}=C^{-\frac{1}{2}}*C^{-\frac{1}{2}}Ac^{2}q^{2}$$

where *Ac* is the correlation distance and *C* is a constant.

Analyzing fractal characteristic is an important method to obtain the microstructure of the nanocomposite interface. The majority of nanomaterials exhibit fractal to a certain level, including the surface fractal and the mass fractal. In Figure 4c, the double logarithmic curve is in a linear region with lnq being −3~0.5. By fitting the linear region using Equation (4), the slope and fractal dimension are obtained, which can be regarded as the quantitative parameters of the irregular degree in the interaction of PI and MWNT.

(4) $$\ln I(0)=\ln I_{0}-\alpha \ln q$$

where *I*(0) is the scattering intensity at *q* = 0. *α* is the negative value of slope, and *α* is in the range of 0–4. If *α* is between 3 and 4, the material belongs to surface fractal when the surface fractal dimension D_s_ = 6 − *α*. If *α* is between 0 and 3, it refers to the mass fractal when the mass fractal dimension D_m_ = *α*. The fractal dimension D reflects the aggregation state and density level of interface. The smaller the fractal dimension is, the better the regularity of interface [33]. From the double logarithmic curves of three nanocomposites, the slopes are −3.50, −3.85, and −3.91 respectively. Then the corresponding fractal dimension can be calculated as 2.50, 2.15, and 2.09 for MWNT/BPDA-ODA, MWNT/BPDA-PDA and MWNT/BTDA-MPD, separately. It indicates that the interface of MWNT/BTDA-MPD is the most regularly arranged, which can be attributed to the interfacial interactions between CNT and PI. According to the molecular dynamics simulation, π–π stacking dominates in the interfacial region of CNT/polyimide nanocomposite [17,18]. Therefore, an assumption can be proposed that the low fractal dimension reflects a more regularly arranged interface, which may be attributed to a high degree of π–π stacking, that is, MWNT/BTDA-MPD, with the lowest fractal dimension, demonstrates a higher π–π stacking effect. 

### 3.2. Phase Dimension Analysis of MWNT/PI Nanocomposites

The one-dimensional correlation function is equivalent to the inverse Fourier transform of scattering intensity functions. Based on the law of electron cloud density of a multiphase system, a correlation function method is utilized for long-period signal analysis (Equation (5)) [34,35], which is plotted with γ(*z*) corresponding with *z*.

(5) $$\lambda (z)=\int I(q)q^{2}\cos(qz)dq$$

**Figure 5:**
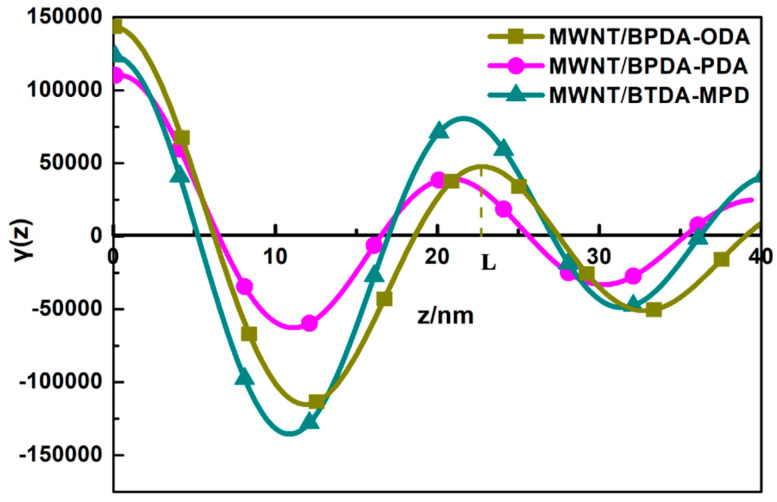
One-dimension electron density correlation function of three aligned MWNT/PI composites.

**Figure 6:**
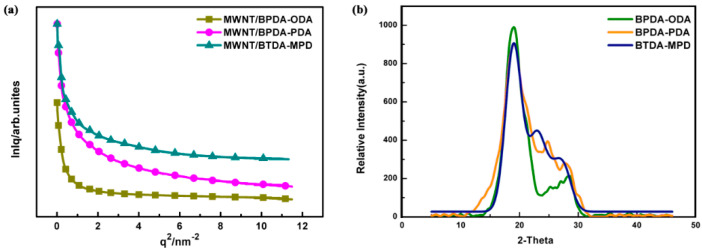
(**a**) Guinier law analysis of scattering curve of the aligned MWNT/PI nanocomposites. (**b**) X-ray diffraction (XRD) diagram of PI with three different molecular structures.

In Figure 5, the one-dimension electron density correlation function shows that all of the three aligned MWNT/PI nanocomposites have a multiphase system that is accompanied by a periodic arrangement structure due to the electron density difference. Additionally, *z* is the correlation distance in the equatorial direction, γ(*z*) is the correlation function of *z*, L is long period length, and dc, dt, da represent different phase dimensions that were extracted using the method mentioned in ref. [36]. The corresponding values obtained from Figure 6 are summarized in Table 1. In order to verify how dc, dt, and da correspond to the dimension of CNT, PI, and interface. Further analysis is conducted in the following part.

By means of Guinier law [37,38], the shape of scatterer, characteristic scale, and distribution in the nonuniform area can be analyzed with the Equation (6). The scattering intensity in the small *q* region is dependent on the radius of gyration (*Rg*).

(6) $$\ln I(q)=\ln I(0)-\frac{1}{3}q^{2}Rg^{2}$$

The linear part in Figure 6a with a small *q* value is used for fitting. The ln*I*(*q*) of scattering intensity and the scattering vector *q*^2^ are highly linear-correlated. The slope of the linear part can be used to calculate the *Rg* that is a shape parameter describing the root mean square of each electron and its center of mass in a material, which can be used to represent the statistical size of scatterer size. The curves in Figure 6a do not show a straight part between 0~2.5 nm^−2^, which suggests that the scatterer is nonspherical and features a polydispersed system [37]. Moreover, the *Rg* of scatterer is not a constant value but with a certain distribution because *Rg* curves are correlated with the slope of the curve. Based on the slope, the *Rg* of MWNT/BPDA-ODA, MWNT/BPDA-PDA and MWNT/BTDA-MPD are 2.42 nm, 2.34 nm and 2.22 nm. Due to the finding from Figure 3, the scatterer causing the long-period signals is attributed to the interface. Therefore, *Rg* of 2.42 nm, 2.34 nm, and 2.22 nm represent the interface distance of MWNT/BPDA-ODA, MWNT/BPDA-PDA, and MWNT/BTDA-MPD. When compared with the dt value in Table 1, it can be found that dt is twice over *Rg* since X-ray is the incident ray propagating along the thickness direction of the nanocomposites, and the penetrating interface is met twice for each period. Then dt/2 is the interface phase dimension. dc is close to 10 nm, which is the diameter of CNT, hence dc represents the CNT phase dimension. 

**Figure 7:**
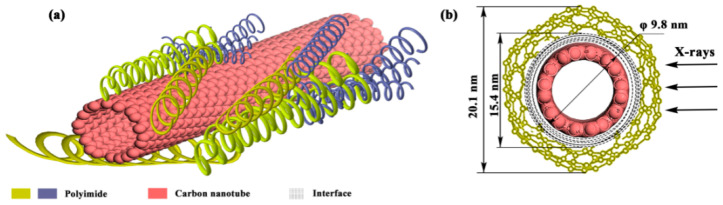
Diagram of MWNT/PI nanocomposites of (**a**) a MWNT wrapped in PI long molecular chains in a period and (**b**) the cross section of nanocomposite when X-ray incident from the side direction.

**Figure 8:**
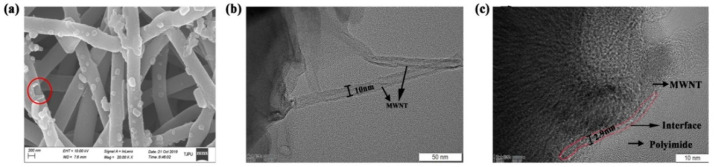
(**a**) SEM image of MWNT/PI composites. HRTEM image of (**b**) aligned MWCNT and (**c**) MWNT/PI composites.

In order to know the relations between da and polyimide phase dimension, XRD was conducted to evaluate the three types of PI. Figure 6b shows that, at 2θ = 18.83° (d = 0.471 nm), there are the main diffraction peaks, which are generated by the parallel-arranged PI layers. The presence of a significant characteristic peak at 2θ = 18.83–27° indicates that the structure of PI is a semicrystalline structure with amorphous regions. The main diffraction peaks are located with preferred orientation of the crystals. The average crystal size is 3.8 nm for BPDA-ODA composites, 3.3 nm for BPDA-PDA composites, and 3.25 nm for BTDA-MPD composites using the Scherrer formula. It can be observed that the da value in Table 1 is also twice over the grain size of PI. This is reasonable because, in the SAXS measurement, the X-ray penetrates PI twice for each period during incidence. The nanocomposites are composed of stacked PI that enwraps MWNTs due to the π–π stacking. Take MWNT/BPDA-ODA as an example to establish a nanocomposite model, shown in Figure 7. The diameter of MWNT is 9.8 nm and the interface distance between MWNT and PI is 2.8 nm. To further verify the model accuracy, HRTEM images of MWNT/ BPDA-ODA nanocomposites are presented in Figure 8. By observing a small portion of PI falling off the MWNT in Figure 6a, it can be confirmed that MWNT is evenly wrapped in PI with a coating structure. The diameter of MWNT is approximately 10 nm and the interface distance is 2.9 nm from Figure 6b,c, which are results consistent with the SAXS characterization results.

### 3.3. π–π Stacking Effect in MWNT/PI Nanocomposite Interface

**Figure 9:**
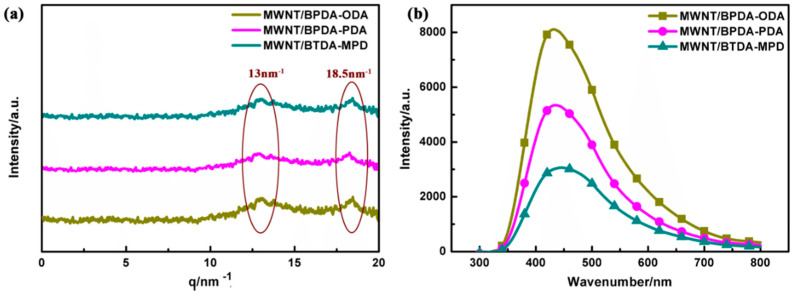
(**a**) Wide-angle X-ray diffraction (WAXD) patterns of the aligned MWNT/PI composites at room temperature and (**b**) the intensity of the fluorescence emission spectra of the aligned MWNT/PI composites.

The walls of carbon nanotubes are composed of hexatomic rings in which each carbon atom undergoes sp^2^ hybridization. sp^2^ hybridization leads to highly delocalized π electrons structure, which subsequently renders the strong interaction between carbon nanotube and polymers with aromatic rings and fused rings [23,39]. WAXD analysis was used to examine the interaction between CNT and PI. Figure 9a shows there are two different characteristic peaks at the range of 0–20 nm^−1^, that is, 13 nm^−1^ and 18.5 nm^−1^. It has been proven that aromatic PI chains, which are oriented in the film plane, are more densely packed along the direction of film thickness rather than along the film plane. Therefore, the presence of 13 nm^−1^ is attributed to the chain stacking of PI [40]. Moreover, there is also a characteristic peak at 18.5 nm^−1^, which suggests that π–π stacking occurs between the hexatomic rings of carbon nanotubes and benzene ring of PI molecular chains [41]. 

To further reveal the interaction between the aligned CNT and PI with π–π stacking (physical crosslinking), a fluorescence spectra test was performed to study the intensity of π–π stacking for three MWNT/PI systems, shown in Figure 9b. It is shown that the strength of fluorescence spectra differs according to the differences in the PI molecular structure. Hence, the π–π stacking of the three aligned MWNT/PI composites can be ranked from the highest to lowest as MWNT/BTDA-MPD, MWNT/BPDA-PDA, and MWNT/BPDA-ODA. The reason for this phenomenon is that stronger π–π stacking induces larger quenching effect [42,43], leading to lower fluorescence intensity. When deeply considering the relationship between the molecular structure and the π–π stacking effect, it can be observed that the two polyimides (BTDA-MPD and BPDA-PDA) with one benzene ring in diamine show higher π–π stacking than BPDA-ODA with ether bond between benzene rings, which may be attributed to the deflection of the benzene ring plane with the existence of ether bond. Therefore, the stacking of benzene rings in diamines promotes the possibility of formation of π–π stacking. Besides, between BTDA-MPD and BPDA-PDA with same diamine, the different π–π stacking effect originates from the molecular structure of dianhydride. BTDA-MPD has a carbonyl group between benzene rings, while two benzene rings are linked by a single bond in BPDA-PDA. It was proved in Ahn et al.’s work that the carbonyl group linked to the benzene ring induced the formation of π–π stacking [42], which is because the benzene rings linked by the carbonyl group tend to form more parallel stacking. The π–π stacking effect reflected by fluorescence spectra is consistent with fractal dimension analysis and interface distance dt/2 that the MWNT/BPDA-MPD nanocomposite has the most regular arrangement attributed to the π–π stacking, thus showing highest π–π stacking intensity and narrowest interface.

## 4. Conclusions

By conducting the experimental analysis on the interface of aligned MWNT and three PIs with different molecular structures, the interfacial characterization was explored. The SAXS analyses showed that all of the three nanocomposites have a nonideal two-phase structure with a long period. The interface between PI and MWNT is complex, nanoscale structural heterogeneity. By adopting a one-dimensional correlation function, each phase dimension in aligned MWNT/PI nanocomposites was calculated and verified by high-resolution transmission electron microscopy (HRTEM) and X-ray diffraction (XRD). It was verified by fractal dimension that MWNT/BTDA-MPD presented the most regular arrangement due to the π–π stacking effect and had the narrowest interface. The π–π stacking was demonstrated to be an important interaction between MWNT and PI via WAXD and fluorescence emission spectroscopy, and it was influenced by the linkage bond between benzene rings in PIs. Two polyimides (BTDA-MPD and BPDA-PDA) with one benzene ring in diamine show higher π–π stacking than BPDA-ODA with an ether bond between benzene rings, which may be attributed to the deflection of benzene ring plane with the existence of ether bond. BTDA-MPD and BPDA-PDA has same diamine, and the different π–π stacking effect originates from the molecular structure of dianhydride. The carbonyl group in BTDA-MPD induced the formation of π–π stacking because the benzene rings linked by the carbonyl group tend to form more parallel stacking. π–π stacking in the three nanocomposites can be ranked from highest to lowest as MWNT/BTDA-MPD, MWNT/BPDA-PDA, and MWNT/BPDA-ODA, corresponding to interface distance of 2.5, 2.6 and 2.8. The analytic method used in this work can be extended to interfacial investigation of CNT/conjugated polymer nanocomposites in other forms by (1) determining the interfacial features and the fractal characteristics, (2) calculating and verifying the phase dimensions, (3) establishing the relationship between interfacial features and quantitative value of π–π stacking. And this work can also provide theoretical foundation for high performance nanocomposite design.

## Figures and Tables

**Table 1:** Statistical data of one-dimensional correlation function.

Sample	L/nm	dc/nm	dt/nm	da/nm
MWNT/BPDA-ODA	22.7	9.8	5.6	7.3
MWNT/BPDA-PDA	21.2	9.7	5.2	6.3
MWNT/BTDA-MPD	22.1	10.4	5.0	6.7
